# Supplementary material for: Molecular Fingerprint of BMD Patients Lacking a Portion in the Rod Domain of Dystrophin
Source: Int J Mol Sci. 2022 Feb 27;23(5):2624. doi: 10.3390/ijms23052624 (PMC8910510; doi:10.3390/ijms23052624)

Supplementary Figure S1. Full-lenght immunoblot images. Vastus lateralis muscle protein extracts (50 μg) from 4 DMD, 4 BMD1 and 4 BMD2 patients were resolved on 10% and 8-12% gradient polyacrylamide gels. Band intensities were normalized against the total amount of proteins stained by Sypro ruby total-protein stain.

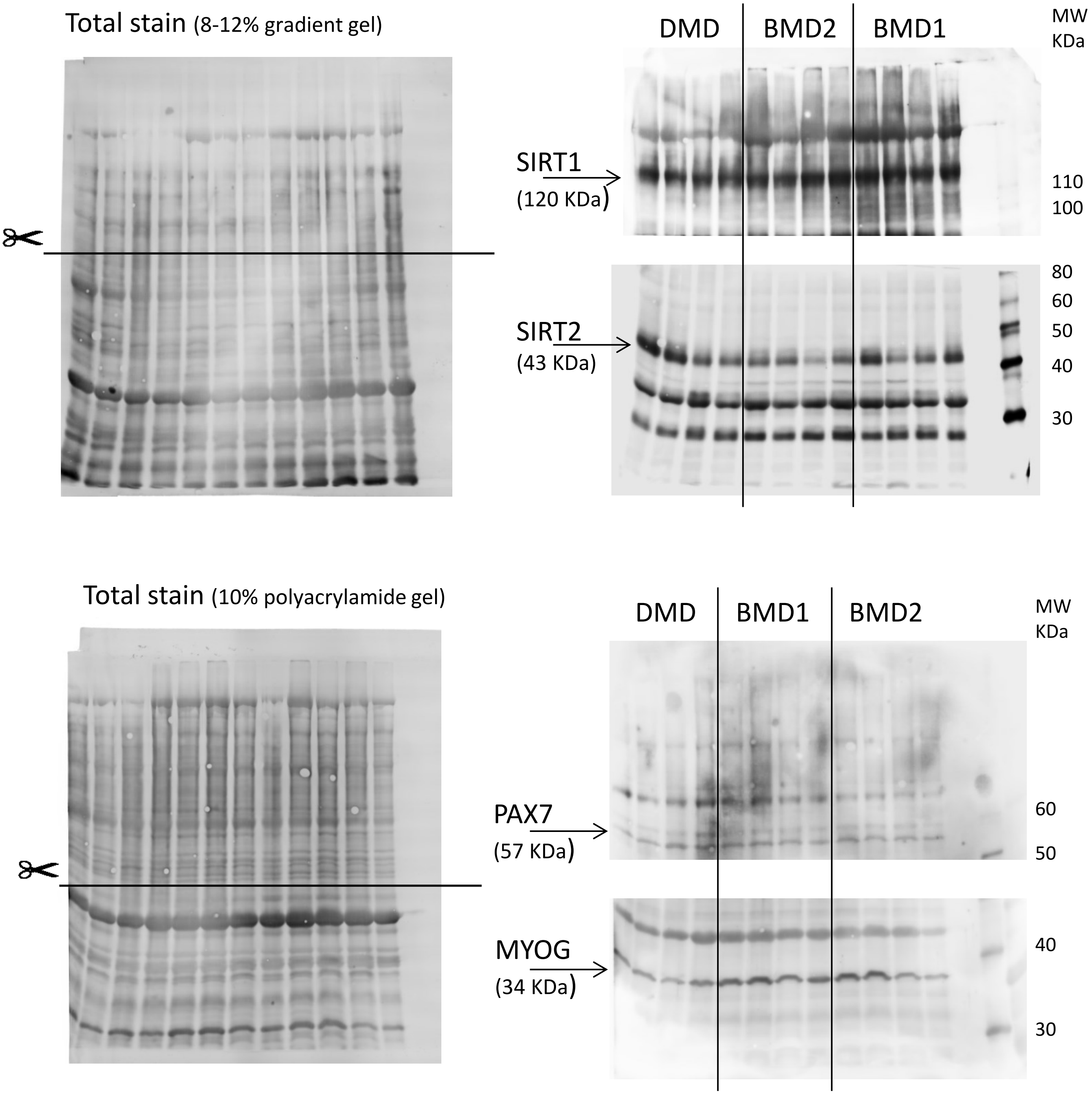

Supplement: Supplementary file 1 [file ijms-23-02624-s001.zip › Supplementary Figure S1.pdf]
